# Enzastaurin inhibits tumours sensitive and resistant to anti-EGFR drugs

**DOI:** 10.1038/sj.bjc.6604493

**Published:** 2008-07-29

**Authors:** T Gelardi, R Caputo, V Damiano, G Daniele, S Pepe, F Ciardiello, M Lahn, R Bianco, G Tortora

**Affiliations:** 1 Dipartimento di Endocrinologia e Oncologia Molecolare e Clinica, Università di Napoli Federico II, Napoli, Italy; 2 Dipartimento Medico-Chirurgico di Internistica Clinica e Sperimentale, Seconda Università di Napoli, Napoli, Italy; 3 Eli-Lilly and Company, Indianapolis, IN, USA

**Keywords:** targeted therapy, EGFR inhibitors, drug resistance

## Abstract

We investigated the antitumour effect and ability to overcome the resistance to anti-EGFR drugs of enzastaurin, an inhibitor of VEGFR-dependent PKC*β* signalling. Enzastaurin was evaluated alone and in combination with the EGFR inhibitor gefitinib, on growth and signalling protein expression in human cancer cells sensitive and resistant to anti-EGFR drugs, both *in vitro* and in nude mice. We demonstrated the marked inhibitory activity of enzastaurin against GEO colon and PC3 prostate cancer cells and their gefitinib-resistant counterparts GEO-GR and PC3-GR, accompanied by inhibition of pAkt and its effector pp70S6K, pGSK3*β* and VEGF expression and secretion. Moreover, enzastaurin showed a cooperative effect with gefitinib in parental and in gefitinib-resistant cells. Remarkably, these results were confirmed *in vivo*, where enzastaurin showed antitumour activity and cooperativity with gefitinib in mice grafted with GEO and GEO-GR tumours, incrementing their median survival and inhibiting the aforesaid protein expression and secretion in tumour specimens. In conclusion, enzastaurin by interfering with signalling proteins implicated in EGFR drug resistance markedly cooperates with gefitinib in sensitive and gefitinib-resistant tumours, thus overcoming and reverting such resistance and providing a rational basis for its development in patients resistant to anti-EGFR drugs.

The epidermal growth factor receptor (EGFR) is a member of the ErbB/HER family of receptor tyrosine kinases (TK), frequently overexpressed in human tumours and directly implicated in the control of cell growth, apoptosis and angiogenesis (Hynes and Lane, 2005). Epidermal growth factor receptor blockade by monoclonal antibodies or small molecule TK inhibitors (TKIs), such as gefitinib or erlotinib, has now entered clinical practice in patients affected by different types of cancer (Mendelsohn and Baselga, 2006; Ciardiello and Tortora, 2008). However, this therapeutic approach has limitations due to the constitutive and/or acquired resistance to EGFR inhibitors, which represents an important emerging problem in cancer treatment. Several groups, including ours, have previously shown that colon tumours that acquire resistance to the anti-EGFR drugs cetuximab or gefitinib exhibit an activation of alternative downstream signal transduction pathways, including increased activity and/or overexpression of pMAPK and VEGF (Viloria-Petit *et al*, 2001; Ciardiello *et al*, 2004; Morgillo and Lee, 2005). Therefore, novel agents that are able to block the above signalling proteins are needed and may be effective also in preventing and/or overcoming the resistance to EGFR inhibitors. A number of inhibitors of VEGF or VEGF receptors (VEGFRs) have already entered clinical practice (Shojaei and Ferrara, 2007), whereas agents targeting signal transducers of the VEGFR pathway are much fewer and are still under evaluation.

The protein kinase C (PKC), particularly the PKC*β* isoform, is involved in VEGFR signal transduction (Xia *et al*, 1996) and can lead to the overproduction of VEGF, as well as to the inhibition of apoptotic cell death (Yoshiji *et al*, 1999; Tsai *et al*, 2003). For all these reasons, PKC*β* is recognised as a significant target for cancer chemotherapy (Hofmann, 2004; Yan Liu *et al*, 2004). The acyclic bisindolylmaleimide LY317615 (enzastaurin HCl) is a potent and selective serine/threonine kinase inhibitor, initially developed as an antiangiogenic adenosine triphosphatase-competitive selective inhibitor of PKC*β* (Faul *et al*, 2003). Enzastaurin dramatically suppressed the growth of new vasculature towards a VEGF-impregnated disc implanted in the rat corneal micropocket (Teicher *et al*, 2002) and decreased microvessel density and plasma VEGF levels in human tumour xenografts (Keyes *et al*, 2004; Graff *et al*, 2005). Enzastaurin has also been tested in combination with radiotherapy in glioma cells (Tabatabai *et al*, 2007). In animal models, enzastaurin showed antitumour and antiangiogenic activity in a wide array of human tumours, including non-small cell lung (NSCL), colon, renal cell and hepatocellular carcinomas (Keyes *et al*, 2002; Liu *et al*, 2004). Enzastaurin has entered clinical evaluation showing good tolerability, manageable side effects and significant activity in patients with lymphomas (Carducci *et al*, 2006) and pretreated high-grade glioblastomas (Fine *et al*, 2005). Enzastaurin has also been tested in combination with gemcitabine and cisplatin and with capecitabine in patients with advanced cancer (Beerepoot *et al*, 2007; Leong *et al*, 2008).

Preclinical and clinical studies suggest that enzastaurin may have an activity broader than PKC*β* inhibition. Moreover, we have hypothesised that its antiangiogenic effects may be exploited to antagonise the resistance to anti-EGFR drugs. Therefore, in the present study, we have evaluated whether enzastaurin is able to inhibit the growth *in vitro* and in nude mice of a variety of human tumours with different degree of expression of EGFR and PKC*β*, including those with acquired or spontaneous resistance to the EGFR inhibitor gefitinib, correlating the effect with the expression of proteins involved in the acquisition of resistance to anti-EGFR drugs. Finally, we have investigated whether enzastaurin is able to revert the resistance to gefitinib.

## Materials and methods

### Drugs

Gefitinib was kindly provided by AstraZeneca Pharmaceuticals (Macclesfield, UK). Enzastaurin was kindly provided by Ely Lilly (Indianapolis, IN, USA).

### Cell cultures

Human GEO colon, PC3 hormone-refractory prostate, MDA-MB-468 breast, SKLU-1 and GLC-82 non-small lung, and PACA44, PANC1 and HPAF pancreatic cancer cells were obtained from the American Type Culture Collection (Manassas, VA, USA). GEO-GR (gefitinib-resistant), GEO-CR (cetuximab-resistant) and PC3-GR (gefitinib-resistant) cells were established as previously described (Ciardiello *et al*, 2004). The resulting cell lines that were stably resistant to EGFR inhibitors retained a resistant phenotype even after several passages in the absence of EGFR antagonists. All cell lines were cultured as previously described (Ciardiello *et al*, 2004).

### Growth in soft agar and analysis of combination index

On day 0, cells were suspended in 0.3% Difco Noble agar (Difco, Detroit, MI, USA) supplemented with complete culture medium, layered over 0.5 ml of 0.8% agar medium base and treated with different concentrations of enzastaurin and gefitinib alone or in combination. After 10–14 days, cells were stained with nitro blue tetrazolium (Sigma Chemical Co., Milan, Italy), and colonies >0.05 mm were counted (Ciardiello *et al*, 2001). Assessment of synergy was performed following the method described by Chou and Talalay (1984) and using the Calcusyn software program (Biosoft, Cambridge, UK). According to this method, combination index (CI) values of <1, 1 and >1 indicate synergy, additivity and antagonism, respectively.

### Cell survival assay

Cells were grown in 24-well plates in the presence of enzastaurin (0.5–2.5 *μ*M). After removing supernatant, 1 mg ml^−1^ of 3-(4,5-dimethylthiazol-2-yl)-2,5-diphenyltetrazolium bromide (MTT; Sigma-Aldrich, Milan, Italy) solution in medium was added to each well. After adding isopropanol, absorbance was measured at 570 nm.

### Western blot analysis

Total cell lysates were obtained from cells cultured *in vitro* or from homogenised tumour. The protein extracts were resolved by 4–15% SDS–PAGE and probed with anti-human, polyclonal Akt, monoclonal pAkt, (Cell Signaling Technologies, Beverly, MA, USA), monoclonal actin (Sigma-Aldrich, Milan, Italy), polyclonal p70S6K and polyclonal pp70S6K (Santa Cruz Biotechnology, CA, USA), monoclonal VEGF, polyclonal pGSK3*β* and polyclonal GSK3*β* (Cell Signaling Technologies). Immunoreactive proteins were visualised by enhanced chemiluminescence (Pierce, Rockford, IL, USA), as described previously (Ciardiello *et al*, 2001).

### Apoptosis detection in cultured cells

The induction of apoptosis was measured using the Cell Death Detection ELISA Plus Kit (Roche Molecular Biochemicals, Mannheim, Germany) (Ciardiello *et al*, 2004). Briefly, cells (5 × 10^4^ cells per well) were seeded into six-multiwell cluster dishes and treated on days 1–2 with enzastaurin (1 and 2.5 *μ*M). Each treatment was performed in quadruplicate. Absorbance readings at 405 nm were normalised for cell number, and the ratio of absorbance of treated cells/untreated cells was defined as the apoptotic index (AI).

### Cell cycle distribution

Cell cycles were measured using YO-PRO-1/propidium iodide (Molecular Probes, Eugene, OR, USA). Cells stained with YO-PRO-1 and propidium iodide were analysed by flow cytometry. After overnight incubation, the cells were exposed to the following treatments at days 1 and 2: enzastaurin 1 and 2.5 *μ*M. After each treatment, cells and medium were collected, washed once in PBS and aliquoted. For cell cycle analysis, cells were resuspended in Krishan's stain and allowed to incubate at 4°C for a minimum of 6 h before analysis. Cell cycle data analysis was performed using the CELL-FIT software (Becton Dickinson, San Jose, CA, USA).

### ELISA assay

The concentrations of hVEGF and murine serum VEGF (mVEGF) in protein extracts or conditioned media from tumour cell lines treated with enzastaurin 1 *μ*M for 48 h were determined by ELISA, as previously described (Errico *et al*, 2004). The absorbance was measured at 490 nm on a microplate reader (Bio-Rad, Hercules, CA, USA). VEGF concentrations were determined by interpolation of the standard curve using linear regression analysis.

### Xenografts in nude mice

Five-week-old Balb/cAnNCrlBR athymic (nu+/nu+) mice (Charles River Laboratories, Milan, Italy) were maintained in accordance with institutional guidelines of the University of Naples Animal Care Committee and in accordance with the Declaration of Helsinki. GEO and GEO-GR human colon cancer cells (10^7^ cells per mice) were resuspended in 200 *μ*l of Matrigel (Collaborative Biomedical Products, Bedford, MA, USA) and injected subcutaneously into mice. After 7 days, when the tumours reached a group mean of 100 mm^3^, tumours were detected and groups of 10 mice were randomised to receive the following treatments: intraperitoneal gefitinib at 150 mg kg^−1^ thrice weekly, enzastaurin by gavage twice daily at 75 mg kg^−1^ based on weekly body measurements for each treated group. Tumour volume was measured using the formula *π*/6 × larger diameter × (smaller diameter)^2^ as previously reported (Ciardiello *et al*, 1996). Two mice were killed on day 25 to perform biochemical analysis. All mice were killed when tumour volume reached 2 cm^3^.

### Statistical analysis

The Student's *t*-test was used to evaluate the statistical significance of the results. All reported *P*-values were two-sided. All analyses were performed with the BMDP New System statistical package version 1.0 for Microsoft Windows (BMDP Statistical Software, Los Angeles, CA, USA).

## Results

### Effect of enzastaurin on the growth of human cancer cell lines with a different degree of sensitivity or resistance to EGFR inhibitors

To test the antiproliferative effect of enzastaurin on cancer cell growth, we treated with different doses of enzastaurin several human cancer cell lines growing in soft agar with established differential sensitivity to EGFR inhibitors, including colon GEO, prostate PC3 and breast MDA-468 cells. GEO cells express low levels of EGFR and are sensitive to both cetuximab and gefitinib, whereas GEO-CR and GEO-GR are highly resistant to both drugs. PC3 possesses similar levels of EGFR and similar sensitivity to gefitinib compared to GEO, opposite to PC3-GR, which are gefitinib-resistant. MDA-MB-468 express high levels of EGFR, yet they are relatively resistant to gefitinib (Bianco *et al*, 2003) and cetuximab (up to 20 *μ*g ml^−1^). GEO cells and derivatives express the wild-type functional form of PTEN, whereas it is deleted and mutated in PC3 and MDA-468 cells, respectively.

**Figure 1:**
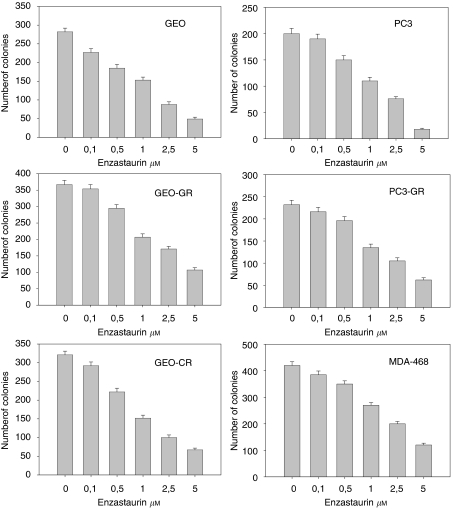
GEO, GEO-CR, GEO-GR, PC3, PC3-GR and MDA-468 cells were treated with enzastaurin at doses of drug ranged from 0.1 to 5 *μ*M. Data are expressed as the number of colony formation. Data represent the average of triplicate determinations of at least two experiments.

Enzastaurin caused a dose-dependent inhibition of colony formation with an IC_50_ of approximately 1 *μ*M for all cancer cell lines tested (Figure 1). In MTT cell survival assay, enzastaurin caused a similar effect to that obtained in the soft agar assay, with an IC_50_ of 1 *μ*M for all cancer cell lines tested. We also tested the effect of enzastaurin treatment on tumour growth in other EGFR-expressing cancer cell lines, including SKLU-1 and GLC-82 (NSCL cancer) and PACA44, PANC1 and HPAF (pancreatic cancer cells). Enzastaurin caused a dose-dependent inhibition of colony formation with an IC_50_ of 1 *μ*M for PACA44, PANC1 and GLC82, and 0.5–1 *μ*M for HPAF and SKLU-1.

We evaluated if the enzastaurin-induced antiproliferative effect was accompanied by induction of programmed cell death. We observed a dose-dependent increase in apoptosis in all human cancer cell lines treated with enzastaurin, as the treatment caused an increase in the AI of 1.5- to 2.5-fold compared with untreated cells. We performed a cell cycle distribution analysis after enzastaurin treatment in all cancer cells, and we observed no perturbations (data not shown).

### Enzastaurin inhibits the PI3K-dependent pathway and reduces the VEGF levels in cell lysate and conditioned medium

**Figure 2:**
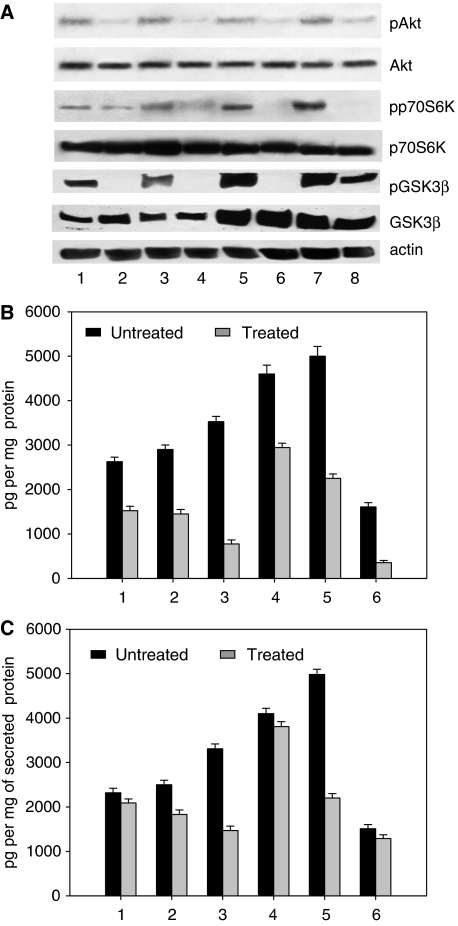
(**A**) Lane 1, GEO cells untreated; lane 2, GEO cells treated with enzastaurin; lane 3, GEO-GR cells untreated; lane 4, GEO-GR cells treated with enzastaurin; lane 5, PC3 cells untreated; lane 6, PC3 cells treated with enzastaurin; lane 7, PC3-GR cells untreated; lane 8, PC3-GR cells treated with enzastaurin. Cell lysates treated *in vitro* on days 0 and 2 were collected on day 5. Bars, s.d. (**B**) ELISA assay for VEGF was done on total lysates from human cancer cell lines treated with 1 *μ*M enzastaurin for 2 days. Lane 1, GEO; lane 2, GEO-CR; lane 3, GEO-GR; lane 4, PC3; lane 5, PC3-GR; lane 6, MDA-468. (**C**) ELISA assay for VEGF was done on conditioned medium collected from the same cell lines. Data are the average of two different experiments, each performed in triplicate; bars, s.d. Results were reported as pg per mg protein.

We evaluated by western blot analysis the effect of treatment on the expression of proteins involved in the PI3K pathway. We observed that enzastaurin had no effect on total Akt, p70S6K and GSK3*β* levels, whereas it markedly inhibited the activated pAkt, its effector pp70S6K and pGSK3*β* levels (Figure 2A).

To investigate the effects of enzastaurin treatment on the production of the main angiogenic growth factor VEGF, the cell lysate and the conditioned medium obtained from the various cell lines treated with enzastaurin were collected and analysed for the presence of VEGF by ELISA assay. As shown in Figure 2B and C, an inhibition of the expression and secretion of VEGF was observed in all cancer cell lines tested, particularly in the EGFR inhibitor-resistant cells.

### Combination treatment of enzastaurin and gefitinib shows a synergism of action in cancer cell lines

**Figure 3:**
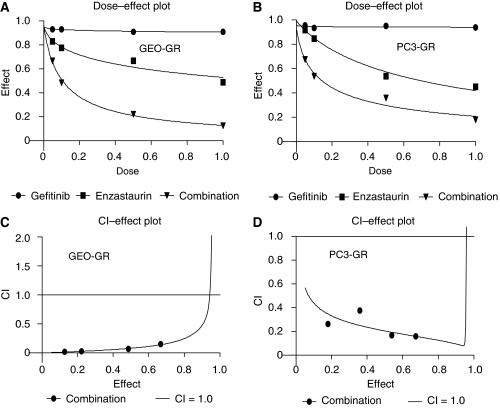
(**A** and **B**) Effect of enzastaurin and gefitinib, alone and in combination, on the soft agar growth of GEO-GR and PC3-GR cells. Growth inhibition results are expressed as the percentage of the number of colonies developed in each of the different treatment wells compared with the absolute number of colonies developed in the untreated control group. Data represent the average of at least two different experiments run in triplicate. (**C** and **D**) Synergistic effect of enzastaurin and gefitinib in combination on GEO-GR and PC3-GR cell growth inhibition. The data represent the plot of CIs, a quantitative measure of the degree of combination treatment for a given end point of the inhibition effect. The CI values of <1, 1 and >1 indicate synergy, additivity and antagonism, respectively. Each point is the mean of at least three different replicate experiments.

We evaluated whether enzastaurin and gefitinib in combination were able to cooperate in inhibiting human cancer cell growth of GEO, GEO-GR, PC3 and PC3-GR. We selected different doses of enzastaurin (0.05–1 *μ*M) and gefitinib (0.05–1 *μ*M) and used them alone and in combination. We used different schedules of administration (enzastaurin followed by gefitinib *vs* gefitinib followed by enzastaurin *vs* simultaneous enzastaurin and gefitinib) and found that the simultaneous administration was the most efficient to inhibit the tumour growth. The effects of drugs, alone and in combination at fixed molar ratios, according to the method of Chou and Talalay (1984), are summarised in the dose–response fit curves generated (Figure 3A and B). To better evaluate the interaction and the possible cooperation between enzastaurin and gefitinib, we performed a combination analysis at their equipotent ratio and generated CI and isobologram curves, according to Chou and Talalay (1984), using an automated calculation software. Values of CI<1 indicate synergism. The combination caused a synergism of action on the soft agar growth in all tested cell lines. In parental GEO and PC3 cells, enzastaurin in combination with gefitinib had a synergistic effect on growth inhibition, particularly with lower doses (data not shown). Interestingly, we observed that in gefitinib-resistant cancer cell lines, GEO-GR and PC3-GR, enzastaurin reverted the resistance to gefitinib. In fact, Figure 3C and D demonstrates that the combination treatment caused a synergistic inhibition of colony formation also in these resistant cancer cells.

### Enzastaurin combined with gefitinib causes potent antitumour activity in xenografted nude mice

**Figure 4:**
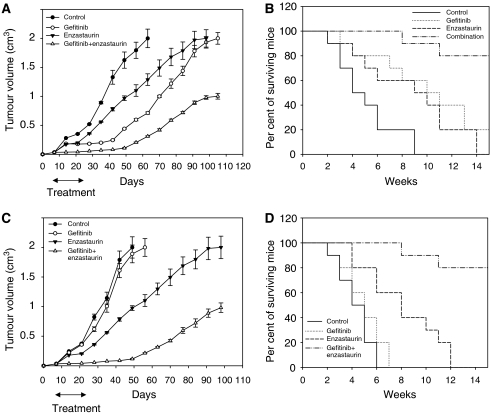
Cooperative effect of enzastaurin and gefitinib on tumour growth and survival of mice bearing human colon cancer xenografts GEO (**A** and **B**) and GEO-GR (**C** and **D**). (**A**–**C**) After 7 days following tumour injection, 10 mice were randomised to receive the treatment. The Student's *t*-test was used to compare tumour sizes among different treatment groups at day 56 following cell injection. Statistically significant differences were observed for enzastaurin *vs* control (two-sided *P*<0.0001), enzastaurin+gefitinib *vs* control (two-sided *P*<0.0001) and *vs* enzastaurin (two-sided *P*<0.0001). Bars, s.d. (**B**–**D**) Median survival in GEO (**B**) and GEO-GR (**D**) xenografts.

BALB/c nude mice xenografted with GEO tumours were treated with enzastaurin and gefitinib, alone and in combination (Figure 4). On day 63, 9 weeks after tumour injection, all untreated mice reached the maximum allowed tumour size of 2 cm^3^. Enzastaurin or gefitinib caused inhibition of tumour growth in mice bearing GEO xenografts. When enzastaurin and gefitinib were used in combination, a potent cooperative antitumour activity was observed. Comparison of tumour sizes among different treatment groups evaluated by the Student's *t*-test was statistically significant (Figure 4A). As compared to 5 weeks median survival in control mice, the survival of mice treated with enzastaurin or gefitinib was 9 and 10 weeks, respectively (Figure 4B). Enzastaurin plus gefitinib did not reach a median survival, because at the end of the experiments, 80% of mice were still alive.

We treated with enzastaurin and gefitinib, alone and in combination, also BALB/c nude mice xenografted with GEO-GR tumours. This tumours grew more aggressively than GEO wild-type, achieving the maximum allowed tumour size of 2 cm^3^ in 6 weeks compared to the 9 weeks of GEO control group. Mice bearing GEO-GR tumours treated with enzastaurin alone reached this size 14 weeks after tumour injection, showing ∼50% growth inhibition, as compared with control after 7 weeks. As expected, treatment with gefitinib did not inhibit the growth of these gefitinib-resistant tumours. Enzastaurin plus gefitinib in combination caused a remarkable cooperative antitumour activity, resulting in a tumour size of only 1 cm^3^ 13 weeks after treatment start. No relevant treatment-related side effects were observed. Comparison of tumour sizes among different treatment groups evaluated by the Student's *t*-test was statistically significant (Figure 4C). The median survival was 8 weeks in enzastaurin-treated group and 5 weeks in control mice (Figure 4D). Enzastaurin plus gefitinib did not reach a median survival, because 80% of mice were still alive at the end of the experiments.

### Combination of enzastaurin and gefitinib inhibits the expression of signalling proteins and VEGF in GEO-GR xenografts

**Figure 5:**
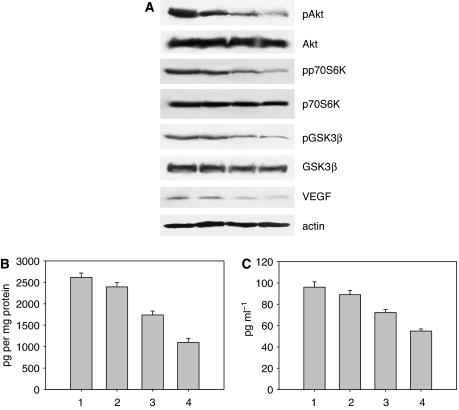
(**A**) Western blotting was performed on total lysates from tumour specimens of two mice of GEO-GR killed on day 25 and treated as in (**C**). Lane 1, untreated control; lane 2, gefitinib; lane 3, enzastaurin; lane 4, gefitinib plus enzastaurin. (**B** and **C**) ELISA assays for hVEGF were performed on total lysates from tumour specimens (**B**) and on serum (**C**) of two mice of GEO-GR killed on day 25.

We analysed the effect of treatment on the expression of a variety of proteins playing a critical role in cancer cell proliferation and angiogenesis in GEO-GR tumours. Western blotting analysis was performed on cell lysates from tumours removed at the end of the third week of treatment, on day 25. As shown in Figure 5A, enzastaurin did not affect the total amount of Akt, p70S6K and GSK3*β*, but inhibited their activated forms pAkt, pp70S6K and pGSK3*β*, and inhibited VEGF expression. Gefitinib did not affect the expression of all signalling proteins observed. When the two agents were used in combination, a more potent inhibition on protein expression was observed.

### Combination of enzastaurin and gefitinib reduces the levels of human VEGF in GEO-GR tumour specimens and mice serum

To evaluate the effect of treatment on VEGF levels, we performed ELISA assays on protein extracts from tumour specimens (Figure 5B) and on serum of GEO-GR-bearing mice (Figure 5C). Enzastaurin reduced the levels of VEGF both in the tumour extracts and in the serum, whereas gefitinib alone had a modest inhibitory activity. The combination of the two drugs markedly inhibited the VEGF production. On the contrary, neither single agent nor their combination affected mVEGF as compared with untreated mice (data not shown).

## Discussion

The EGFR pathway is also implicated in several intracellular processes that control growth and angiogenesis. Monoclonal antibodies or TKIs targeting EGFR are today used in clinical practice (Mendelsohn and Baselga, 2006; Ciardiello and Tortora, 2008). However, molecular changes in EGFR-dependent or EGFR-independent signalling pathways can lead to resistance to EGFR inhibitors (Morgillo and Lee, 2005; Ono and Kuwano, 2006). In fact, cancer cells may acquire resistance to EGFR inhibitors by increasing the expression and/or the activity of several signalling proteins downstream to EGFR, including pMAPK, pAkt and VEGF, which represent the major escape pathways for EGFR inhibition (Ciardiello *et al*, 2004; Zhang *et al*, 2004; Morgillo and Lee, 2005). These same resistance mechanisms occur in GEO-GR and PC3-GR cells as compared to their parental counterparts (Ciardiello *et al*, 2004; present work and unpublished observations). We and others have shown on these bases the therapeutic advantage of a simultaneous blockade of EGFR and VEGF/VEGFRs (Ciardiello *et al*, 2000, 2004; Kerbel and Folkman, 2002; Tortora *et al*, 2008). Therefore, novel agents able to inhibit Akt and VEGF pathways may represent an important therapeutic tool and they may also help in preventing the occurrence of resistance to EGFR inhibitors.

In this context, PKC*β*, which activates the PI3K/Akt pathway (Aeder *et al*, 2004; Kawakami *et al*, 2004) and transduces VEGFR signalling, may be a valuable target. Enzastaurin is a novel inhibitor of PKC*β* that interferes with angiogenesis and cell proliferation and has shown good tolerability and activity in clinical studies in glioblastoma and lymphoma (Fine *et al*, 2005; Carducci *et al*, 2006).

In the present study, we have investigated the effect on growth and protein expression of enzastaurin, used alone and in combination with the EGFR TKI gefitinib, in human cancer cells sensitive or resistant to gefitinib, *in vitro* and in nude mice.

We have shown that enzastaurin alone has an antiproliferative effect on several human cancer cell lines EGFR inhibitor-sensitive and EGFR inhibitor-resistant. Biochemical analysis demonstrated that enzastaurin inhibits the expression of pAkt in all of the cancer cell lines examined and suppresses the expression and secretion of VEGF, especially in those cell lines resistant to EGFR inhibitors. In addition, enzastaurin efficiently inhibits pGSK3*β*, a PKC and Akt downstream target, and pp70S6K another Akt effector. Interestingly, the degree of PKC*β* expression does not influence the therapeutic efficacy of enzastaurin. We then investigated the effect of enzastaurin in combination with gefitinib in cancer cell lines, and we demonstrated a synergistic growth inhibitory effect in GEO and PC3 cells, evident also with suboptimal doses of enzastaurin. Interestingly, a synergism of action was observed also in the gefitinib-resistant GEO-GR and PC3-GR cells, in which single-agent gefitinib was totally ineffective. These results demonstrate that enzastaurin, by targeting critical proteins such as pAkt and VEGF pathways, is not only active when used alone, but also can abrogate the resistance to EGFR inhibitors, strictly depending on those proteins.

We translated this strategy *in vivo*, in nude mice bearing sensitive and gefitinib-resistant GEO and GEO-GR colon tumours, respectively. In GEO tumours, treatment with enzastaurin or gefitinib caused an inhibition of tumour growth, and combination of two drugs had a potent cooperative effect. In GEO-GR tumours, gefitinib, as expected, was ineffective whereas enzastaurin alone caused a significant inhibition of tumour growth. Enzastaurin in combination with gefitinib revealed a marked antitumour effect, resulting in 80% of mice still alive at the end of the experiments. Moreover, a western blot analysis performed on tumour specimens to evaluate the effect of treatment on protein expression revealed that, whereas gefitinib alone was ineffective, enzastaurin was able to inhibit pAkt, pp70S6K, pGSK3*β* and VEGF, and the combination of the two agents caused a more efficient inhibition of the above proteins. ELISA assay of tumour and blood samples confirmed that the combination treatment inhibited the expression and the secretion of VEGF.

Taken together, all the above data demonstrate that enzastaurin is active on tumour proliferation and angiogenesis of cancer cells sensitive and resistant to EGFR inhibitors. Importantly, enzastaurin has a cooperative effect with gefitinib and is able to overcome and revert the resistance to gefitinib in cancer cells resistant to this anti-EGFR drug, likely due to the ability of enzastaurin to inhibit the Akt and VEGF pathways, directly responsible for the escape mechanisms activated in tumours resistant to EGFR inhibitors. Therefore, enzastaurin in combination with anti-EGFR drugs may be proposed as a novel therapeutic strategy worth further investigation in a clinical setting.
